# Compressive stress inhibits proliferation in AsPC-1 pancreatic cancer cells and reduction of Myc protein

**DOI:** 10.1371/journal.pone.0352769

**Published:** 2026-07-09

**Authors:** Fuko Miyake, Seiichiro Ishihara, Naomi Ando, Shoichiro Tange, Atsushi Enomoto, Hisashi Haga

**Affiliations:** 1 Division of Soft Matter, Graduate School of Life Science, Hokkaido University, Sapporo, Hokkaido, Japan; 2 Department of Advanced Transdisciplinary Sciences, Faculty of Advanced Life Science, Hokkaido University, Sapporo, Hokkaido, Japan; 3 Department of Biological Sciences, Graduate School of Science, Osaka University, Osaka, Japan; 4 Institute for Protein Research, Osaka University, Osaka, Japan; 5 Division of Medical Genome Sciences, Department of Genomic and Preventive Medicine, Sapporo Medical University School of Medicine, Sapporo, Hokkaido, Japan; 6 Department of Pathology, Nagoya University Graduate School of Medicine, Nagoya, Aichi, Japan; University of Kentucky, UNITED STATES OF AMERICA

## Abstract

Cancer cells grow in confined tumor microenvironments and push the surrounding tissue, which reacts and exposes them to compressive stress. Previous studies have revealed that compressive stress inhibits proliferation in breast and colorectal cancer cells. It has been reported that pancreatic tumors accumulate compressive stress *in vivo*, but it is not well understood whether compressive stress regulates proliferation in pancreatic cancer cells. Therefore, the effect of compressive stress on the proliferation of human pancreatic cancer cells was investigated in this study. We found that the growth of compressed AsPC-1 human pancreatic cancer cells was slower than that of uncompressed cells. Furthermore, the protein expression of Myc was reduced in compressed cells, which inhibited their proliferation. In addition, the expression of Snail, which is correlated with poor prognosis in pancreatic cancer patients, was promoted by compressive stress and the inhibition of Myc. These results suggest that compressive stress inhibits proliferation and triggers Snail expression via the reduction of Myc protein in AsPC-1 pancreatic cancer cells.

## Introduction

Cells respond to various stimuli from surrounding extracellular matrix (ECM) and cells [1,2]; these stimuli include biochemical stimuli, such as growth factors and chemokines [2], and mechanical stimuli, such as stiffness of ECM and the pressure of interstitial fluid [1]. As well as biochemical stimuli, mechanical stimuli are critical for cellular phenomena, including growth [3,4], migration [5,6], and differentiation [7,8]. The mechanical environment of a malignant tumor is typically abnormal [9]. In pancreatic tumor, matrix stiffening, microarchitecture remodeling, high interstitial fluid pressure, and high compressive (solid) stress are the major mechanical characteristics that influence tumor progression [10]. Cancer cells have been suggested to sense these mechanical stimuli and regulate disease progression. Indeed, mechanical properties such as ECM stiffness are critical for the progression of various types of cancers [11–14]. However, the effects of mechanical stimuli, particularly compressive stress, on cancer progression are not well understood.

Cancer cells grow in a confined tumor microenvironment and push against surrounding tissue. As a reaction from the surrounding tissue, especially in relation to stiffened ECM within the tumor, cancer cells are exposed to compressive stress [4]. Previous studies have suggested that compressive stress regulates growth [4,15] and migration [16] in cancer cells. It has been reported that compressive stress inhibits cell division during cell growth by G1 arrest via p27 in human breast cancer, human and mouse colon cancer, and human and mouse sarcoma cells [15,17]. However, whether compressive stress regulates proliferation in pancreatic cancer cells has not been clarified.

In this study, we established a method for compressing cultured cells *in vitro* and analyzing the proliferation of pancreatic cancer cells. Our results revealed that the proliferation of AsPC-1 human pancreatic cancer cells was inhibited by compressive stress. We also found that Myc protein, a critical regulator of cell cycle, was downregulated by compressive stress, followed by the phosphorylation of Rb to prevent cell proliferation. Furthermore, the Snail expression was upregulated by compressive stress and Myc inhibitor in AsPC-1 pancreatic cancer cells. These results indicate that compressive stress in tumor suppresses proliferation of pancreatic cancer cells and induces Snail expression via Myc downregulation.

## Materials and methods

### Cell culture

The AsPC-1 human pancreatic adenocarcinoma cells (Female, CRL-1682, American Type Culture Collection) were cultured in the culture media (RPMI 1640 Medium (11875-119, Thermo) supplemented with 10% fetal bovine serum (172012, Sigma Nichirei Biosciences) and 1% antibiotic/antimycotic solution (A5955, Sigma-Aldrich)). Cells were cultured at 37 °C in a humidified incubator with 5% CO_2_. Cells were tested for mycoplasma contamination using a MycoAlert Assay Control Set (LT07-518, Lonza Bioscience) and not authenticated. The following inhibitors were used for cell culture: Myc inhibitor MYCi361 (10 μM, T12132, TargetMol) and CHIR99021 (5, 10, or 20 μM, 038-23101, Wako). Dimethyl Sulfoxide (DMSO, 046-21981, Wako) was used for negative control. Phase-contrast images were captured with a TE300 microscope (Nikon Instech) equipped with a 10x objective.

### *In vitro* compression cell culture

1.5 x 10^5^ to 8 x 10^5^ cells were seeded on 6 well plates or glass dishes with an inner diameter of 34 mm coated with 0.3 mg/mL Cellmatrix Type I-C (Nitta Gelatin). After 24 h, the media was changed to 3 mL of fresh media. An agarose gel (3% PrimeGel Agarose PCR-Sieve HRS 150 bp-800 bp (TAKARA) in Elix water, ~ 2.3 mm thickness, 33–34 mm diameter, UV treated) was incubated with the culture media for 24 h and added to the well or dish. A stainless weight (illustrated in S1 Fig) coated with silicone (SH9555, DOW) was placed on the agarose gel. The holes of the weight enable us to capture phase-contrast images of the compressed cells. For the control, a sample without a weight was prepared. The cells were cultured for ~3 days with or without compression. The media was changed every day. In this system, the expression of most hypoxia-inducible factor 1-induced genes [18] did not increase significantly after compression, suggesting that the compression system did not induce hypoxia. For the analysis of cell volume and height, the cells cultured on a glass dish were stained with Calcein-AM (1 μM, Wako) for 1 h. Three-dimensional (3D) images of the cells were then captured using an A1 confocal laser scanning microscope (Nikon Instech) equipped with a 60x objective. After compression for 2, 7, or 24 h, 3D images of the cells were also captured. For cells compressed for 7 or 24 h, 1 μM Calcein-AM was added 1 h before capturing the images. The cell volume and height were analyzed using ImageJ software (National Institute of Health).

### Cell growth assay

After culturing the cells on 6 well plates with or without compression, the agarose gel and weight were removed, and the media in the dish was transferred to a centrifuge tube. The cells were treated with 0.25% Trypsin-EDTA (25200−072, Gibco) for 8 min at 37 °C. The cells were suspended and transferred to the same tube. The cells in the dish were washed with 1 mL culture media and collected to the same tube, and the mixture in the tube was centrifuged at 1,000 rpm for 2 min. After removing the supernatant, the cell pellet was suspended with 1 mL of culture media. The number of cells was counted with Countess I FL (Thermo).

### Immunofluorescent staining

After culturing the cells on glass dishes with or without compression, the agarose gel, weight, and media were removed. The cells were then washed with phosphate buffered saline (PBS), fixed with 4% paraformaldehyde in PBS for 15 min at 4 °C, and washed thrice with PBS. Next, the cells were permeabilized with 0.3–0.5% Triton X-100 in PBS for 15 min at room temperature and washed thrice with PBS. The cells were blocked with 5% bovine serum albumin (BSA) (for phosphor-Rb) or 5% skimmed milk (for Myc) in PBS for 60 min at room temperature. After PBS wash, a primary antibody solution (1:7200 Anti-phospho-Rb, #8516, Cell Signaling Technology) or (1:10000 Anti-Myc, ab32032, abcam) in PBS was added, and the mixture was incubated at 4 °C overnight. After three washes with PBS, a secondary antibody solution (1:200 Goat anti-Rabbit IgG(H + L), Superclonal Recombinant Secondary Antibody, Alexa Fluor 488, A27034, Invitrogen; 1:4,000 Hoechst 33342, H1399, Invitrogen) in PBS was added, and the mixture was incubated at room temperature for 1–1.5 h. After washing thrice with PBS, fluorescent images were captured using a C2 confocal imaging system (Nikon Instech) with a 60x or 20x objective. To analyze the phospho-Rb intensity in the nuclei, Cellpose (v2.2.3) was used [19]. To quantify the localization of Myc, fluorescence intensity was calculated as the cytoplasm/nuclei ratio using ImageJ software (National Institute of Health). The images for comparison in the same experiment were generated under the same experimental conditions, including sample preparation, microscopy setting, and image analysis.

### Western blotting

After culturing the cells on 6 well plates with or without compression, the agarose gel, weight, and media were removed, and the cells were washed with PBS. Proteins were extracted from the cells using sodium dodecyl sulfate (SDS) sample buffer (0.125 M Tris HCl (pH = 6.8), 2.3% SDS, 10% glycerol, and 5% dithiothreitol, 0.01% bromophenol blue). The samples were then boiled for 5 min. We used 10% (phosphor-Rb, MYC, and β-actin) polyacrylamide gels for SDS-PAGE (20 mA per gel). After SDS-PAGE, blotting to polyvinylidene difluoride membranes was performed (86 mA per gel). After blotting, the membranes were incubated in 5% skim milk (Myc, phosphor-Myc-T58, or β-actin) or 5% BSA (phosphor-Rb) in Tris buffered saline plus Tween 20 (TBS-T) for 60 min at room temperature, and then incubated with primary antibodies (1:20,000, phosphor-Rb, #8516, Cell Signaling Technology; 1:10,000 Anti-Myc, ab32072, abcam; 1:10,000, Anti-phospho-Myc-T58, #46650, Cell Signaling Technology; or 1:50,000 Anti-β-actin, ab6276, abcam) in Can Get Signal Solution 1 (NKB-101, TOYOBO) at 4 °C overnight. After washing thrice with TBS-T, the membranes were incubated with secondary antibodies (Anti-rabbit IgG, HRP-linked Antibody, 7074S, Cell Signaling Technology for phosphor-Rb (1:20,000), Myc (1:10,000), or phosphor-Myc-T58 (1:10,000); or Anti-mouse IgG, HRP-linked Antibody, 7076S, Cell Signaling Technology for β-actin (1:500,000)) in Can Get Signal Solution 2 (NKB-101, TOYOBO) for 1 h at room temperature. After washing thrice with TBS-T, signals were detected using an Immobilon Western Chemiluminescent HRP substrate (WBKLS0500, Millipore) and ChemiDoc Touch Imaging System (BioRad). The relative intensity of proteins was calculated by normalizing the intensity of target proteins with the intensity of β-actin analyzed via Image Lab software (BioRad).

### RNA extraction and qPCR

After culturing the cells on 6 well plates with or without compression, the agarose gel, weight, and media were removed. The cells were then washed with PBS. RNA was extracted using a FastGene RNA Basic Kit (FG-80250, NIPPON Genetics); cDNA synthesis was performed using ReverTra Ace qPCR RT Master Mix (FSQ-201, TOYOBO); Quantitative PCR (qPCR) was performed by KAPA SYBR Fast qPCR kit (KK4602, NIPPON Genetics) and StepOnePlus (Thermo Scientific); and the relative expression of mRNA was calculated by normalizing the expression of target mRNA with the expression of β-actin (*ACTB*). The following primers were used: (5’ to 3’): β-actin (*ACTB*) (Forward: TGGGACGACATGGAGAAAATCTG, Reverse: AGGTCTCAAACATGATCTGGGTC), Myc (*MYC*) (Forward: GCGACTCTGAGGAGGAACAAGAAG, Reverse: GTTGTGCTGATGTGTGGAGACG), p21 (*CDKN1A*) (Forward: ACAGCAGAGGAAGACCATGTG, Reverse: GGTAGAAATCTGTCATGCTGGTC), Snail (*SNAI1*) (Forward: TGGTTCTTCTGCGCTACTGC, Reverse: GCTGCTGGAAGGTAAACTCTGG), Ki67 (*MKI67*) (Forward: ATCGTCCCAGTGGAAGAGTTG, Reverse: TCGACCCCGCTCCTTTTGATAG), *CA9* (Forward: ATGAGAAGGCAGCACAGAAGGG, Reverse: TGAGCAGGACAGGACAGTTACC), *PDK1* (Forward: ACACCATGCCAACAGAGGTG, Reverse: AACCAAAACCAGCCAGAGGC), *PGK1* (Forward: AAGGTTAAAGCCGAGCCAGC, Reverse: TCTGCAACTTTAGCTCCGCC), *VEGFA* (Forward: ATACAGAAACCACGCTGCCG, Reverse: AATTCCAAGAGGGACCGTGC).

### Data analysis of patients

The GEPIA2 (Gene Expression Profiling Interactive Analysis, http://gepia2.cancer-pku.cn/#index) database was used to compare the expression levels of *SNAI1* in pancreatic adenocarcinoma with corresponding normal tissues. Kaplan–Meier curves of overall survival in pancreatic cancer patients were analyzed using the Kaplan–Meier plotter [20].

### Statistical analysis

When comparing a value showing variance to another value with no variance, we determined significance via the 95% confidence interval. To compare between two values with variance, we used the two-sided Student’s t-test, Welch’s t-test, or Wilcoxon rank-sum test to analyze statistical significance. To compare cell volume before and after compression, we used paired t-test to analyze statistical significance. For multiple comparisons, we analyzed significance using the Bonferroni correction. All analyses were performed using Excel software. No statistical methods were performed to pre-determine the appropriateness of sample size.

## Results

### Establishment of *in vitro* compression cell culture system

**Fig 1:**
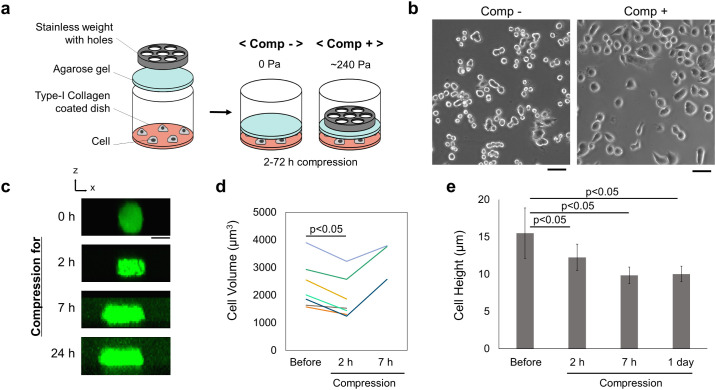
*In vitro* compression cell culture of pancreatic cancer cells. (a) Illustration of *in vitro* compression system for cultured cells. (b) Phase contrast images of AsPC-1 cells without compression (Comp -) or with compression (Comp +) for 24 h. Bar = 20 µm. (c) Three dimensional images of AsPC-1 cells after compression for 0, 2, 7, 24 h. Bar = 10 µm. x and z axis of the images are shown together. (d) Time series changes of the volume in AsPC-1 cells after compression calculated in (c). n = 7 cells. Statistical significance was calculated using paired t-test. (e) Time series changes of the height in AsPC-1 cells after compression calculated in (c). Mean±SD. 22 ≤ n ≤ 37 cells. Statistical significance was calculated using Wilcoxon rank-sum test. Representative analysis of three independent experiments is shown.

To observe an analyze cancer cells, we first established a method for compressing cultured cells *in* *vitro*. Cells on a cultured well or dish were compressed with a stainless weight with holes (Fig 1a, S1a Fig). To compress the cells uniformly, we inserted agarose gel between the cells and the weight (Fig 1a). The cells were exposed to approximately 240 Pa compression, which mimicked the stress within pancreatic tumor tissue [21]. Through this method, we confirmed that AsPC-1 human pancreatic cancer cells, which contain *4-hit* (*KRAS, TP53, CDKN2A,* and *SMAD4*) mutations and exhibit malignant phenotypes [22], exhibited spreading morphology with compression (Fig 1b, S1b Fig). In addition, 3D observations confirmed that the volume and height of AsPC-1 cells decreased after compression for 2 h (Fig 1c, 1d, 1e). After compression for 7 h, the cell volume was recovered in several cells, whereas the height of these cells was still decreased after compression for 7 h or 1 day, suggesting that AsPC-1 cells spread on the dish after compression. Overall, we successfully compressed AsPC-1 cells using an *in vitro* culture system.

### Inhibition of proliferation in AsPC-1 pancreatic cancer cells by compressive stress

**Fig 2:**
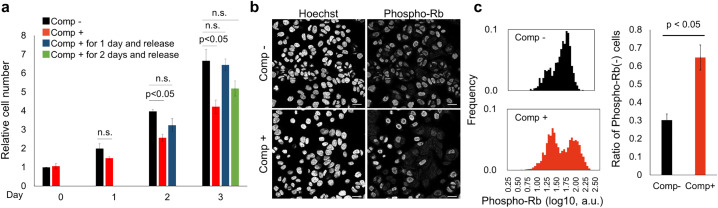
Inhibition of proliferation in AsPC-1 cells after compression. (a) Relative cell number after compression. n = 3 experiments. Mean±SE. Statistical significance was calculated using Student’s t-test. n.s.: not significance, comp + for 1 day and release: the weight was released after compression for 1 day, comp + for 2 day and release: the weight was released after compression for 2 days. (b) Representative fluorescent images of nuclei (Hoechst) and phosphorylated Rb (Phospho-Rb) in AsPC-1 cells without compression (Comp -) or with compression (Comp +) for 3 days. Bar = 20 μm. Representative images from n = 3 experiments are shown. (c) Histogram of fluorescent intensity of phosphorylated Rb (Phospho-Rb) (left) and ratio of Phospho-Rb negative cells (right) in AsPC-1 cells calculated in (b). n = 3 experiments. Mean±SE. Statistical significance was calculated using Student’s t-test.

We next analyzed cell proliferation of AsPC-1 cells with or without compressive stress. We cultured AsPC-1 cells with or without compression for 3 days and found that the increase in cell number was slower in the compressed cells than in the uncompressed cells (Fig 2a). In addition, AsPC-1 cells released from the weight after compression for 1 day recovered proliferation (Fig 2a), suggesting that the inhibition of proliferation in pancreatic cancer cells is reversible. We then analyzed phosphorylated Rb protein, a critical inducer of G1-S transition in the cell cycle [23]. Immunofluorescent staining of phosphorylated Rb revealed that compressive stress significantly increased the phosphorylated Rb-negative cells (Fig 2b, 2c). In contrast, phospho-Rb-positive and phosphor-negative cells were present in the compressed cell population (Fig 2c), indicating that AsPC-1 cells are heterogenous. These results suggest that compressive stress inhibits proliferation of compression-sensitive AsPC-1 pancreatic cancer cells by the suppression of G1-S transition.

### Downregulation of Myc protein in AsPC-1 pancreatic cancer cells by compressive stress

**Fig 3:**
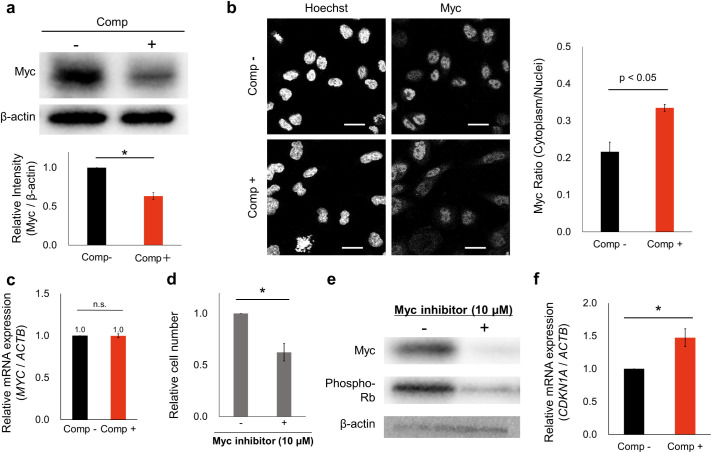
Reduction of Myc protein in AsPC-1 cells after compression. (a) Western blotting of Myc and β-actin in AsPC-1 cells without compression (Comp -) or with compression (Comp +) for 3 days. Relative expression of Myc/β-actin (Mean±SE) is shown together. n = 3 experiments. *statistical significance with 95% confidence interval. (b) Representative fluorescent images of nuclei (Hoechst) and Myc in AsPC-1 cells without compression (Comp -) or with compression (Comp +) for 2 days. Bar = 20 μm. Myc intensity ratio (Cytoplasm/Nuclei, Mean±SE) is shown together. Statistical significance was calculated using Welch’s t-test. n = 3 experiments. 139 ≤ n ≤ 249 cells. (c) qPCR of *MYC* and *ACTB* in AsPC-1 cells without compression (Comp -) or with compression (Comp +) for 3 days. n = 3 experiments. n.s.: no statistical significance with 95% confidence interval. (d) Relative cell number in AsPC-1 cells treated without (-) or with (+) Myc inhibitor (10 μM) for 24 h. n = 2 experiment. Mean±SE. *statistical significance with 95% confidence interval. (e) Western blotting of Myc, phosphorylated Rb (Phospho-Rb), and β-actin in AsPC-1 cells without or with Myc inhibitor (10 μM) for 24 h. (f) qPCR of *CDKN1A* and *ACTB* in AsPC-1 cells without compression (Comp -) or with compression (Comp +) for 3 days. n = 3 experiments. *statistical significance with 95% confidence interval.

Myc is a critical transcription factor for G1-S transition in the cell cycle by the downregulation of p21 (*CDKN1A*) [24]. We therefore investigated whether compressive stress regulates protein expression, localization, and mRNA expression in Myc. Western blotting showed a decrease in Myc protein in AsPC-1 cells following compressive stress (Fig 3a), and the nuclear localization of Myc protein was also reduced following compression in AsPC-1 cells (Fig 3b). However, the mRNA expression of *MYC* was not significantly changed by compression (Fig 3c). These results indicate that Myc protein was degraded after exposing AsPC-1 cells to compressive stress. Previous studies have shown that Myc is stable with Serine 62 (S62) phosphorylation, whereas the ubiquitination of Myc is triggered following phosphorylation of Threonine 58 (T58), which is regulated by ubiquitin-ligase and induces degradation of Myc by proteasome (S2a Fig) [25]. GSK3 is one of the major kinases for Myc phosphorylation [25], and we therefore treated AsPC-1 cells with GSK3 inhibitor CHIR99021 and analyzed Myc protein stability. The results confirmed that CHIR99021 treatment increased Myc protein and decreased phosphorylated Myc at T58 in a dose dependent manner (S2b Fig), suggesting that the amount of Myc protein in AsPC-1 cells is dependent on GSK3-regulated T58 phosphorylation and proteasome degradation.

### Regulation of proliferation in AsPC-1 pancreatic cancer cells by Myc

We then inhibited Myc activity using a Myc inhibitor MYCi361, and analyzed proliferation and Rb phosphorylation in AsPC-1 cells. Myc inhibitor treatment decreased the cell number and amount of phosphorylated Rb in uncompressed AsPC-1 cells (Fig 3d, 3e). Furthermore, mRNA expression of p21 (*CDKN1A*), a transcriptional target of Myc, was promoted by compressive stress in AsPC-1 cells (Fig 3f). In contrast, we confirmed that the inhibition of GSK3 did not increase cell number but increased Myc expression in compressed AsPC-1 cells (S2c and S2d Fig). These results indicate that Myc is essential for cell proliferation whereas in compressed cells, recovering Myc protein alone is insufficient to restore cell proliferation. Therefore, an additional mechanism may exist to inhibit cell proliferation in compressed cells. In addition, the GSK3 inhibitor itself prevents cell growth in AsPC-1 cells [26] and may suppress cell proliferation in addition to restoring Myc expression. Collectively, these results suggest that compressive stress promotes p21 expression through the downregulation of Myc protein, which prevents G1-S transition by the dephosphorylation of Rb and the subsequent inhibition of cell proliferation.

### Induction of snail in AsPC-1 pancreatic cancer cells by compressive stress via Myc downregulation

**Fig 4:**
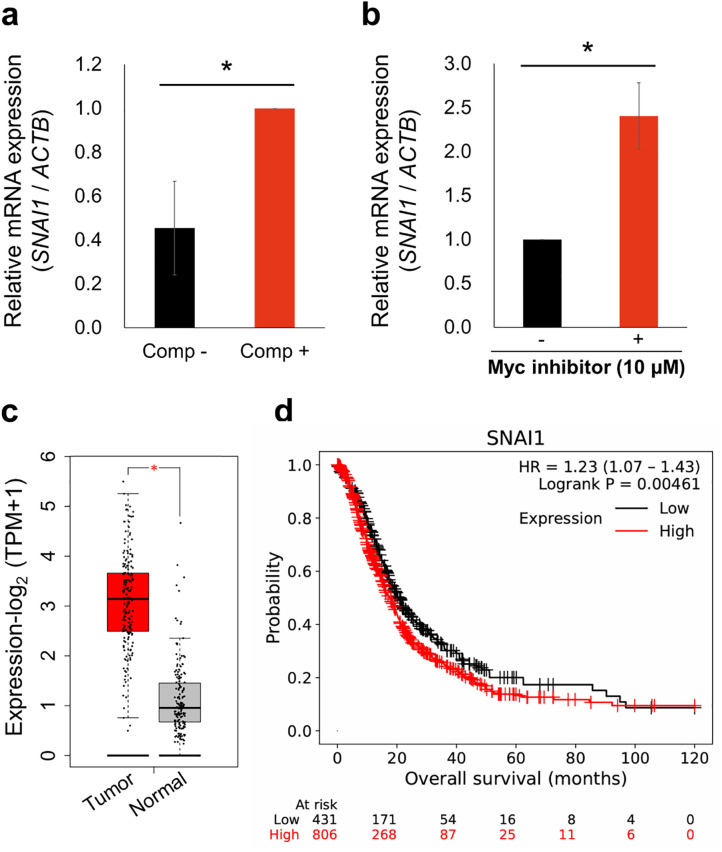
Induction of Snail (*SNAI1*) expression in AsPC-1 cells after compression and Myc inhibition. (a) qPCR of *SNAI1* and *ACTB* in AsPC-1 cells without compression (Comp -) or with compression (Comp +) for 3 days. n = 3 experiments. Mean±SE. *statistical significance with 95% confidence interval. (b) qPCR of *SNAI1* and *ACTB* in AsPC-1 cells treated without (-) or with (+) Myc inhibitor (10 μM) for 24 h. n = 3 experiments. Mean±SE. *statistical significance with 95% confidence interval. (c) *SNAI1* mRNA levels in pancreatic adenocarcinoma with corresponding normal tissues. (d) Kaplan–Meier curves of overall survival in relation to the gene expression of *SNAI1* in pancreatic cancer patients.

Previous studies have reported that Myc inhibition suppresses cell proliferation and induces dormancy [27], which is critical for drug resistance in pancreatic cancer cells [28]. Snail is one of the dormancy-related molecules [29], and it suppresses the cell cycle by inducing hypo-phosphorylation of Rb protein [30]. We therefore evaluated the expression of Snail gene (*SNAI1*) in compressed AsPC-1 cells. *SNAI1* expression was promoted by compressive stress in AsPC-1 cells (Fig 4a), and Myc inhibition enhanced *SNAI1* expression in AsPC-1 cells (Fig 4b). *SNAI1* expression in pancreatic cancer patients was upregulated compared with corresponding normal pancreatic tissues (Fig 4c) and correlated with poor prognosis (Fig 4d). However, proliferation marker Ki67 (*MKI67*) expression was not reduced after compressive stress in AsPC-1 cells (S3a Fig), indicating that compressive stress did not induce G0 arrest or dormancy. Taken together, these results suggest that compressive stress triggers *SNAI1* expression through Myc inhibition, and this is followed by the progression of pancreatic cancer independently on dormancy.

## Discussion

Previous reports have shown that compressive stress prevents cell growth by G1 arrest in colon and breast cancer cells [15,17]; as such, the inhibitory effect of compressive stress on proliferation via G1 arrest may be common in solid cancer cells. Pancreatic cancer is reported to be a compressed tumor [9]; however, the role of compressive stress in pancreatic cancer cells is not well understood. In this study, we established an *in vitro* culture system for cell compression. Through the use of this system, we revealed that compressive stress inhibits proliferation by inducing G1 arrest in AsPC-1 pancreatic cancer cells, which agrees with previously reported results relating to several solid cancer cell types, including breast and colon cancer cells.

The mechanical microenvironment of pancreatic tumors differs from that of normal pancreatic tissues. In pancreatic tumors, matrix stiffening, microarchitecture remodeling, high interstitial fluid pressure, and high compressive (solid) stress are the major mechanical characteristics that influence tumor progression [10]. It has been suggested that pancreatic cancer cells sense these mechanical stimuli and regulate pancreatic cancer progression. In this study, we analyzed the effects of compressive stress on the phenotype of pancreatic cancer cells. Therefore, we established a compressive experimental culture system to minimize the effects of other mechanical stimuli, including matrix stiffness, matrix architecture, and fluid stress, on cancer cells by culturing the cells on collagen-coated plastic or glass dishes. Thus, we suggest that compressive stress is a critical mechanical cue for influencing proliferation in pancreatic cancer cells.

We showed that compressive stress decreases the amount of Myc protein and Myc inhibition suppresses proliferation of AsPC-1 pancreatic cancer cells. Compressive stress also promoted the expression of proliferation suppressor p21, a transcriptional target of Myc. However, the mRNA expression of Myc was not regulated by compressive stress, which suggests that Myc protein is degraded by compressive stress. Previous studies have reported that ubiquitinated Myc is degraded by proteasome [25,31–33] and triggered by phosphorylation in Myc at T58 induced by GSK3 [25]. Indeed, we showed that GSK3 inhibitor suppressed the phosphorylation of Myc at T58 and the degradation of Myc in AsPC-1 cells. Therefore, GSK3 may be activated by compressive stress in pancreatic cancer cells. GSK3 inhibitor is reported to be a good candidate molecule for pancreatic tumor treatment [26]. We suggest that combination therapy involving a GSK3 inhibitor and a present drug such as gemcitabine or nub-paclitaxel may improve pancreatic cancer treatment.

We also revealed that compressive stress and Myc inhibitor induces Snail expression in AsPC-1 pancreatic cancer cells. Snail is reported as a dormancy-relate molecule [29] that suppresses the cell cycle by inducing hypo-phosphorylation of Rb protein [30]. It has been suggested that dormancy in AsPC-1 cells is important for drug resistance and survival [28]. In addition, high Snail expression contributes to drug resistance, metastasis, and stemness in pancreatic cancer cells [34,35]. However, compressive stress did not decrease the expression of the proliferation marker Ki67, indicating that compression did not induce G0 arrest or dormancy. We showed that Snail expression correlates with poor prognosis in pancreatic cancers, suggesting that compressive stress triggers Snail expression by downregulating Myc protein, resulting in increasing malignancy of pancreatic cancer cells and a poor prognosis for pancreatic cancer patients.

**Fig 5:**
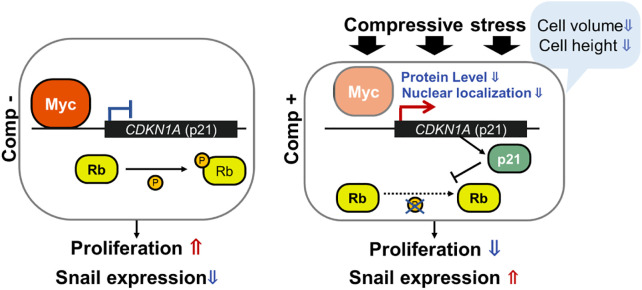
Schematic model of inhibition in proliferation and induction of Snail regulated by compressive stress via Myc downregulation in AsPC-1 pancreatic cancer cells.

In summary, we showed that compressive stress inhibits proliferation and promotes Snail expression via Myc downregulation in AsPC-1 pancreatic cancer cells (Fig 5). Compressive stress reduces cell volume and height, and this is followed by a reduced protein amount and nuclear localization in Myc. Compressive stress also induces the Myc target gene *CDKN1A* (p21), which is critical for G1 arrest by preventing Rb phosphorylation. Compressive stress and Myc inhibition trigger Snail expression, which is related to a poorer pancreatic cancer prognosis. These results indicate that compressive stress affects cellular function in pancreatic cancer cells. As such, drugs that regulate compression-sensitive signaling may be candidates for treating pancreatic cancer. However, the AsPC-1 population contains both compression-sensitive and compression-insensitive cells; thus, the effect of compression stress on pancreatic tumor progression *in vivo* should be further examined in future studies.

## Supporting information

S1 Fig *In vitro* compression system. (a) Detailed illustration of the weight used *in vitro* compression experiments. (b) A phase contrast image of AsPC-1 cells cultured on a collagen-coated plastic dish for 24 h. Bar = 20 µm.
(TIF)

S2 Fig GSK3-dependent Myc degradation in AsPC-1 cells. (a) Schematic model of Myc stability regulated by its phosphorylation. (b) Western blotting of Myc, phosphorylated Myc at T58 (Phospho-Myc-T58), and β-actin in AsPC-1 cells with GSK3 inhibitor CHIR99021 (0, 5, 10, 20 μM). Relative expressions of Myc/β-actin and Phospho-Myc-T58/β-actin (Mean value) are shown together. (c) Relative cell number without (DMSO) or with GSK3 inhibitor CHIR99021 (5 μM) after compression. n = 2 experiments. Mean±SD. Statistical significance was calculated using Student’s t-test. n.s.: not significance. (d) Western blotting of Myc and β-actin in AsPC-1 cells without compression (Comp -) or with compression (Comp +) for 3 days. CHIR990021 (+) cells were treated with GSK3 inhibitor CHIR99021 (5 μM) for 3 hours before analysis. Relative expressions of Myc/β-actin are shown together. n = 2 experiments. Mean±SE. *statistical significance with 95% confidence interval. n.s.: no statistical significance with 95% confidence interval.
(TIF)

S3 Fig Compression does not affect the expression of *MIK67* and hypoxia-induced genes. qPCR of (a) *MKI67* and (b) hypoxia-inducible factor 1-induced genes (*CA9, PDK1, PGK1, and VEGFA*) with *ACTB* in AsPC-1 cells without compression (Comp -) or with compression (Comp +) for 3 days. n = 3 experiments. Mean±SD. n.s.: no statistical significance with 95% confidence interval. *statistical significance with 95% confidence interval.
(TIF)

S1 Table The dataset used to build graphs. (XLSX)

S1 raw images The raw images of western blots for Fig 3a, 3e, and S2b, S2d Fig. (PDF)
