# Supplementary figures and images for: Compressive stress inhibits proliferation in AsPC-1 pancreatic cancer cells and reduction of Myc protein
Source: PLoS One. 2026 Jul 9;21(7):e0352769. doi: 10.1371/journal.pone.0352769 (PMC13349192; doi:10.1371/journal.pone.0352769)

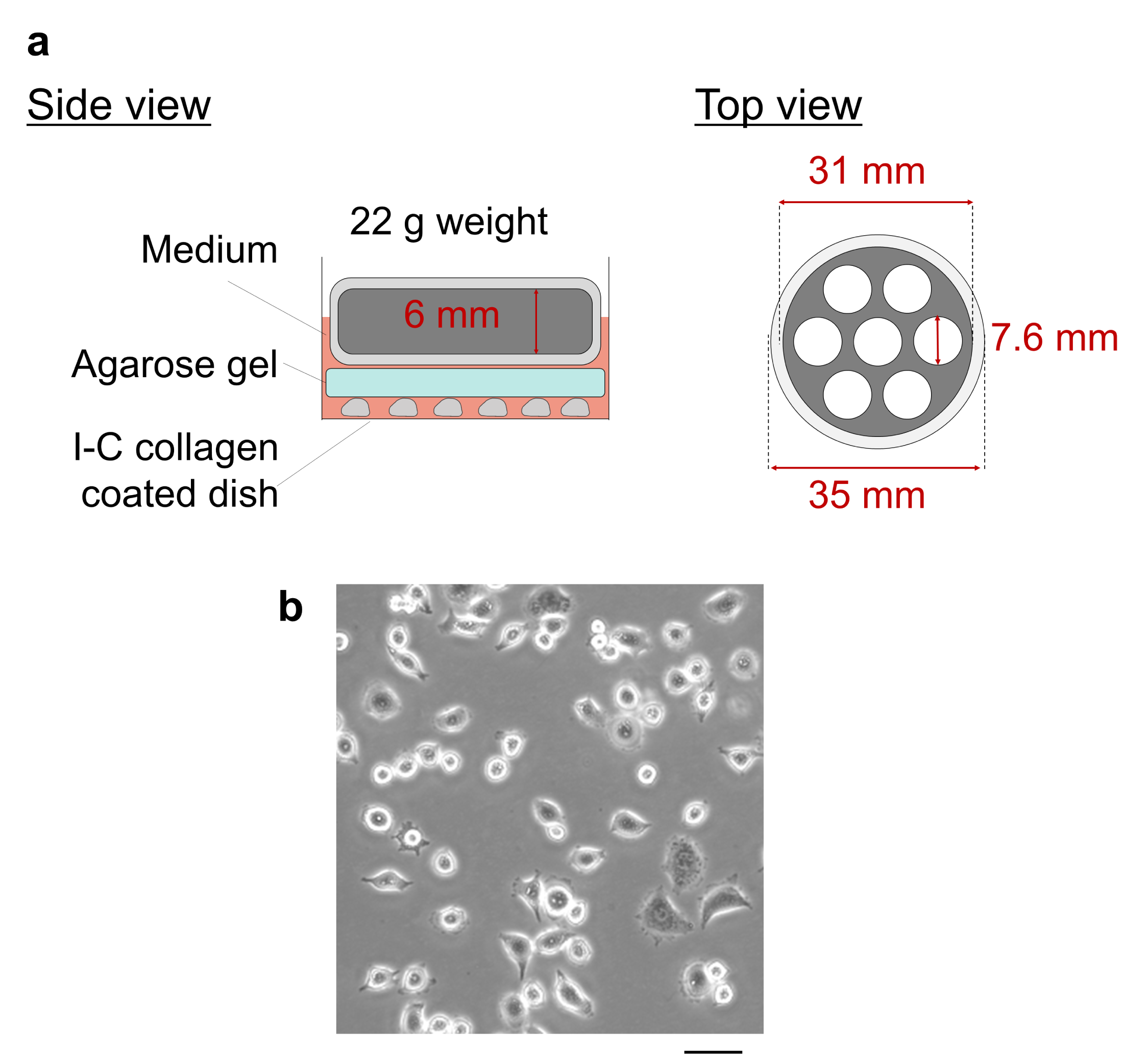

Supplement: S1 Fig — (a) Detailed illustration of the weight used in vitro compression experiments. (b) A phase contrast image of AsPC-1 cells cultured on a collagen-coated plastic dish for 24 h. Bar = 20 µm. (TIF) [file pone.0352769.s001.tif]

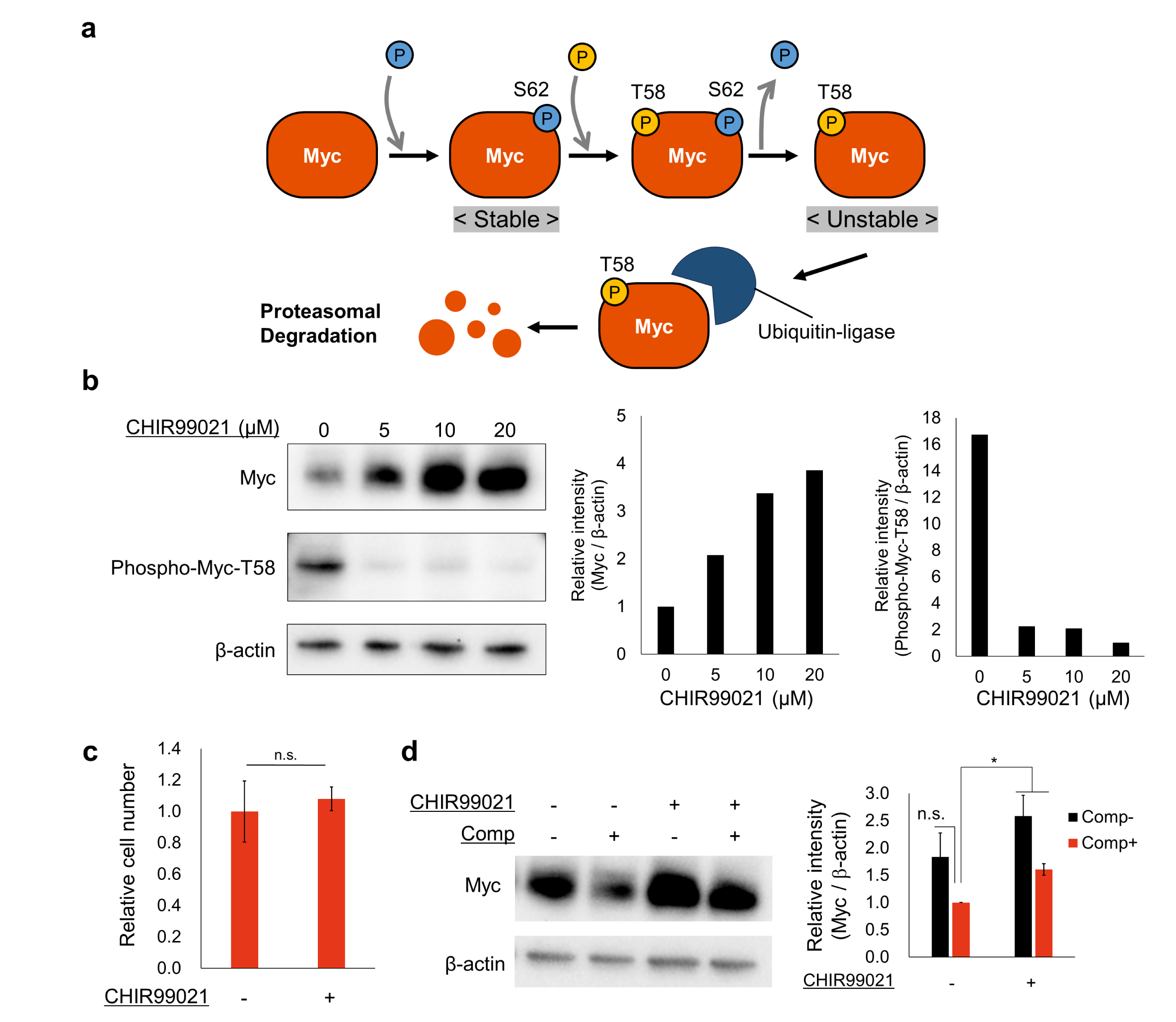

Supplement: S2 Fig — (a) Schematic model of Myc stability regulated by its phosphorylation. (b) Western blotting of Myc, phosphorylated Myc at T58 (Phospho-Myc-T58), and β-actin in AsPC-1 cells with GSK3 inhibitor CHIR99021 (0, 5, 10, 20 μM). Relative expressions of Myc/β-actin and Phospho-Myc-T58/β-actin (Mean value) are shown together. (c) Relative cell number without (DMSO) or with GSK3 inhibitor CHIR99021 (5 μM) after compression. n = 2 experiments. Mean±SD. Statistical significance was calculated using Student’s t-test. n.s.: not significance. (d) Western blotting of Myc and β-actin in AsPC-1 cells without compression (Comp -) or with compression (Comp +) for 3 days. CHIR990021 (+) cells were treated with GSK3 inhibitor CHIR99021 (5 μM) for 3 hours before analysis. Relative expressions of Myc/β-actin are shown together. n = 2 experiments. Mean±SE. *statistical significance with 95% confidence interval. n.s.: no statistical significance with 95% confidence interval. (TIF) [file pone.0352769.s002.tif]

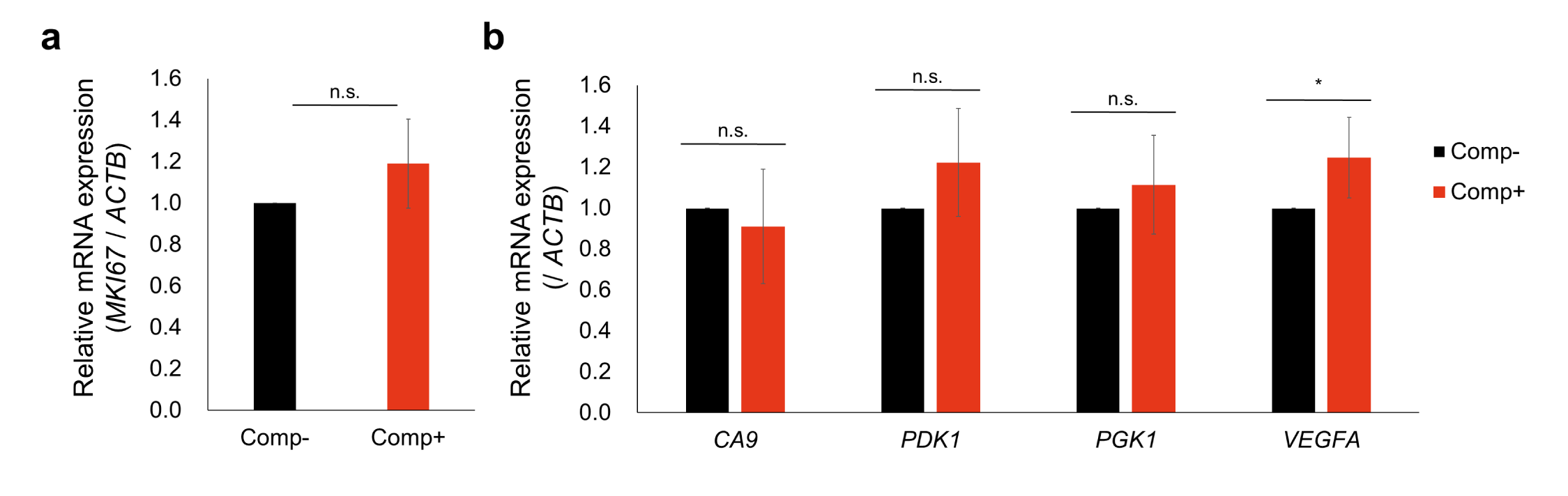

Supplement: S3 Fig — qPCR of (a) MKI67 and (b) hypoxia-inducible factor 1-induced genes (CA9, PDK1, PGK1, and VEGFA) with ACTB in AsPC-1 cells without compression (Comp -) or with compression (Comp +) for 3 days. n = 3 experiments. Mean±SD. n.s.: no statistical significance with 95% confidence interval. *statistical significance with 95% confidence interval. (TIF) [file pone.0352769.s003.tif]
