# Supplementary material for: Compressive stress inhibits proliferation in AsPC-1 pancreatic cancer cells and reduction of Myc protein
Source: PLoS One. 2026 Jul 9;21(7):e0352769. doi: 10.1371/journal.pone.0352769 (PMC13349192; doi:10.1371/journal.pone.0352769)

**Signals were detected using an Immobilon Western Chemiluminescent HRP substrate (WBKLS0500, Millipore) and ChemiDoc Touch Imaging System (BioRad).**

## Fig 3a

Comp

- +

Myc (~62 kDa)

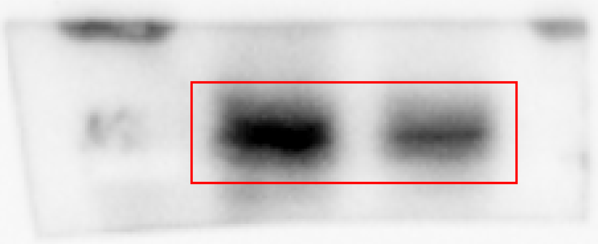

β-actin (~42 kDa)

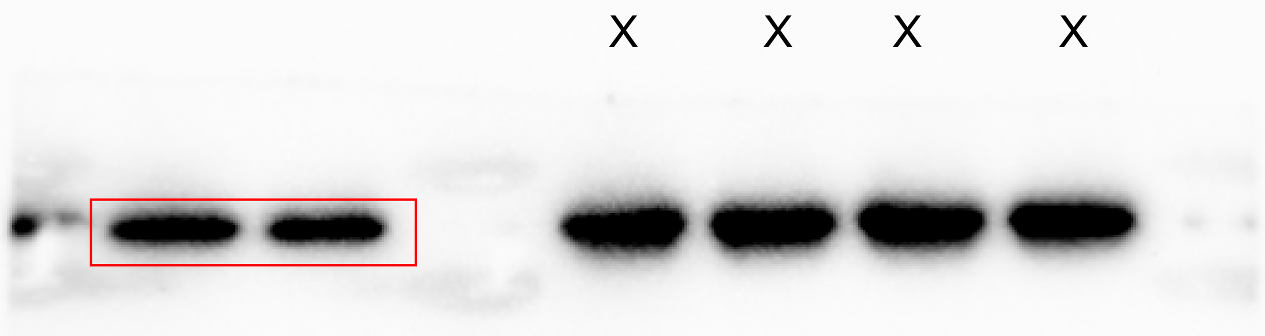

**Signals were detected using an Immobilon Western Chemiluminescent HRP substrate (WBKLS0500, Millipore) and ChemiDoc Touch Imaging System (BioRad)**

## Fig 3e

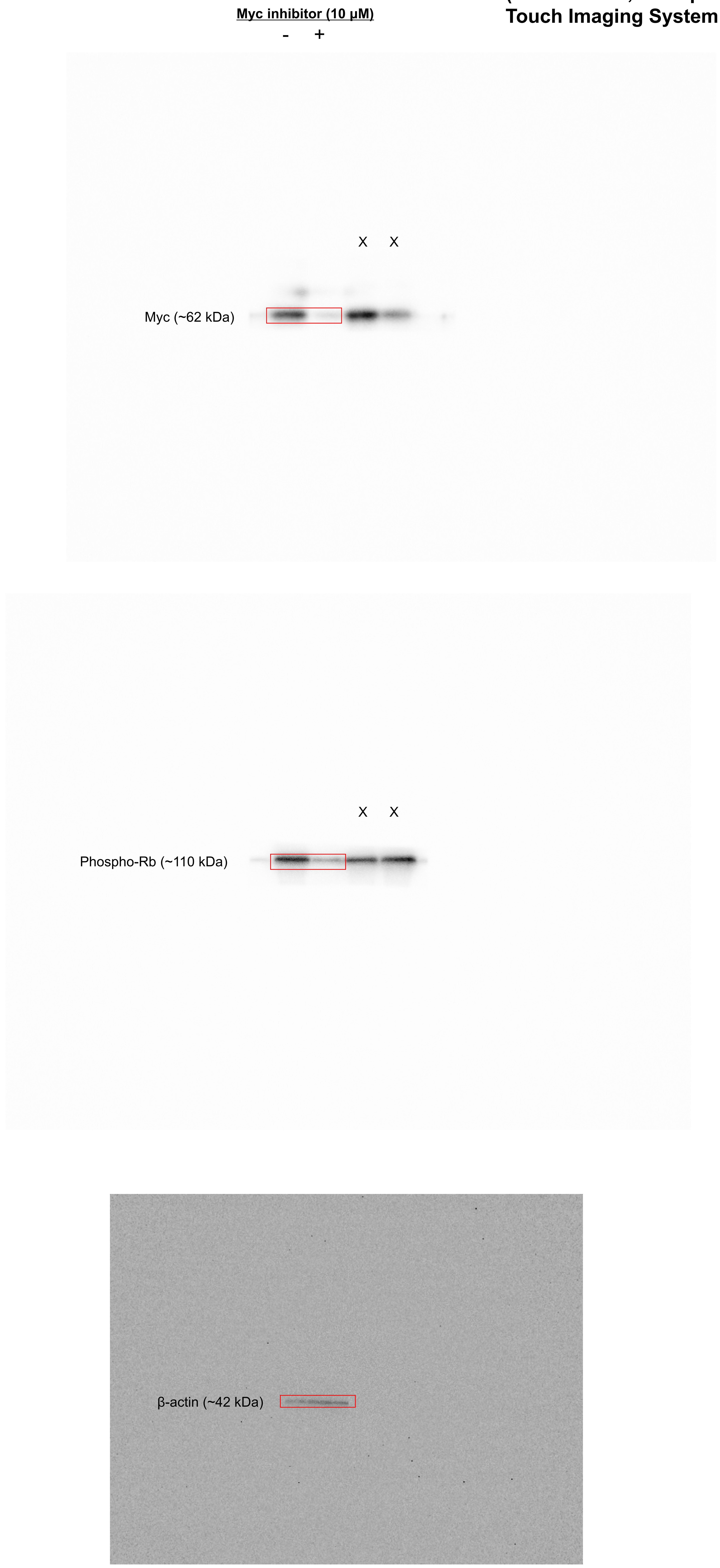

Signals were detected using an Immobilon Western Chemiluminescent HRP substrate (WBKLS0500, Millipore) and ChemiDoc Touch Imaging System (BioRad)

## S2b Fig

CHIR99021 (μM) 0 5 10 20

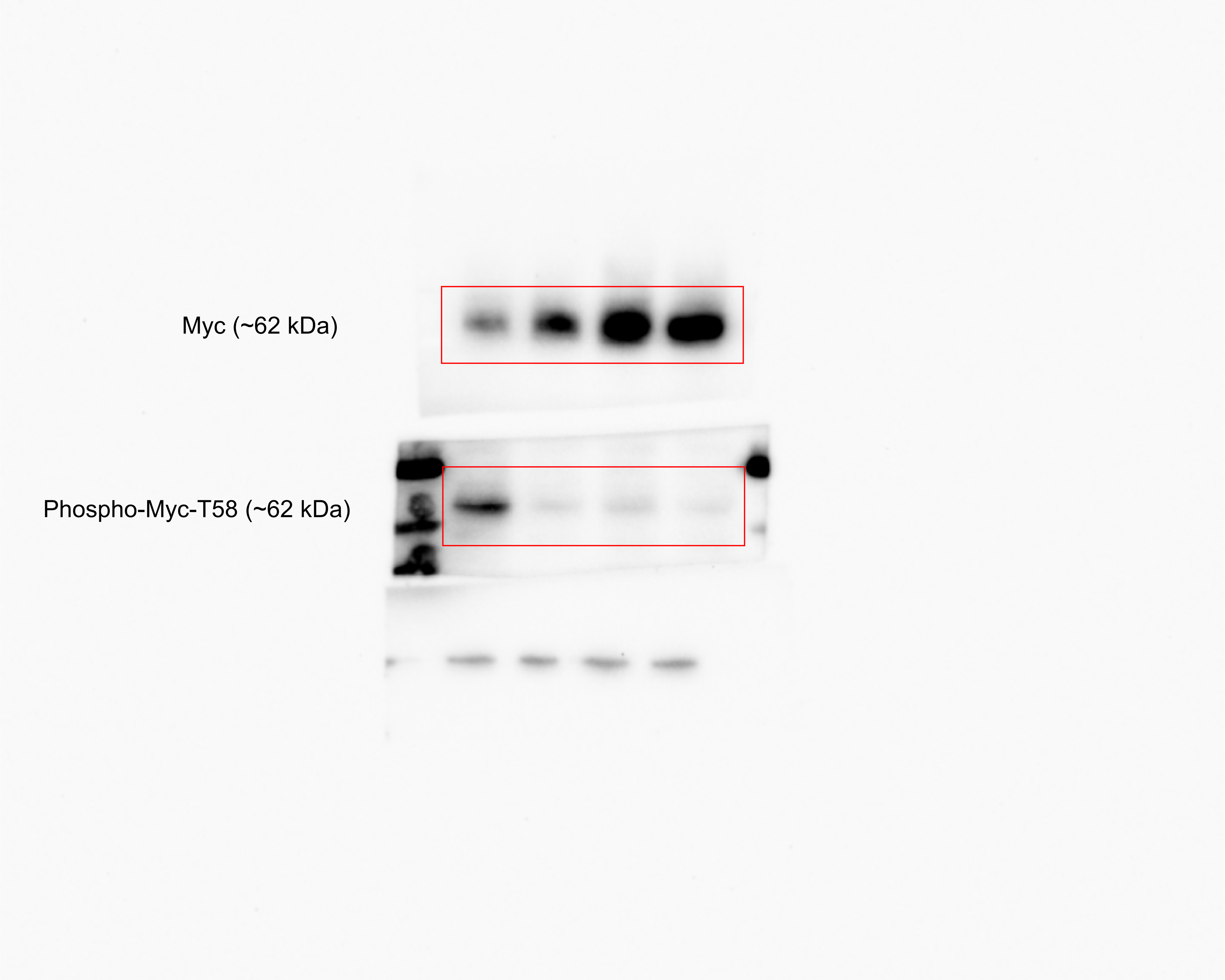


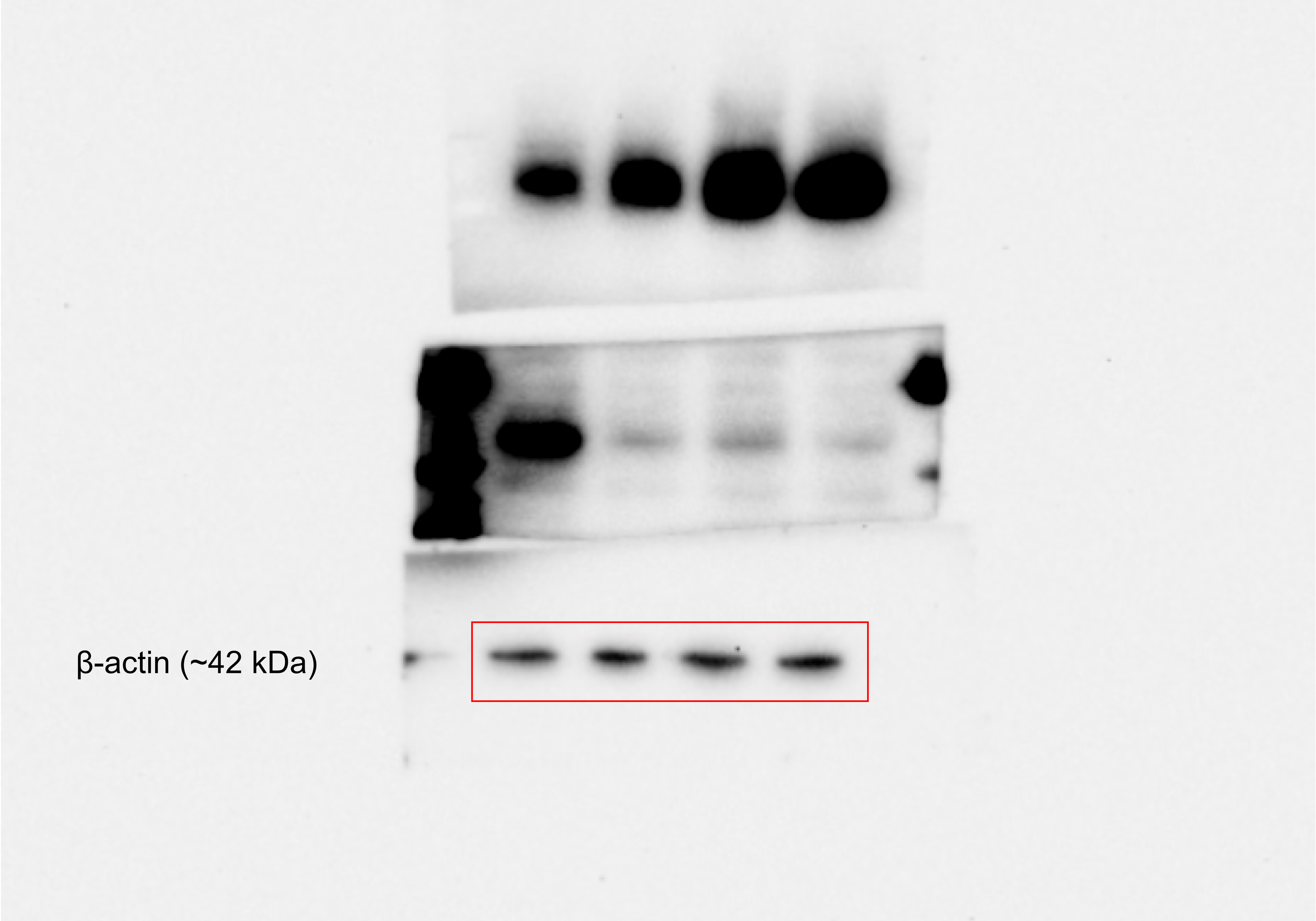

**S2d Fig**

Signals were detected using an Immobilon Western Chemiluminescent HRP substrate (WBKLS0500, Millipore) and ChemiDoc Touch Imaging System (BioRad)

CHIR99021 (μM) 0 5 10 20

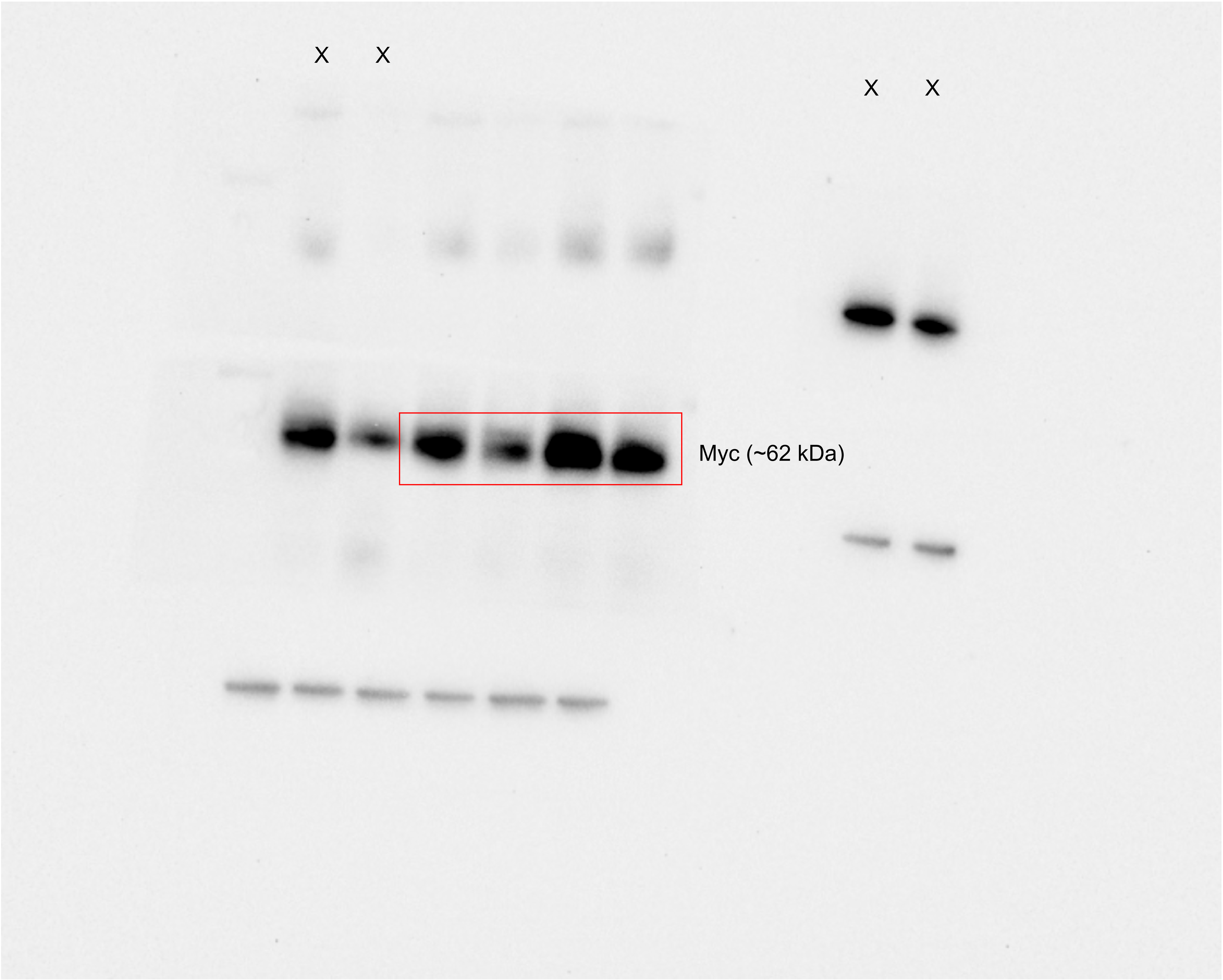


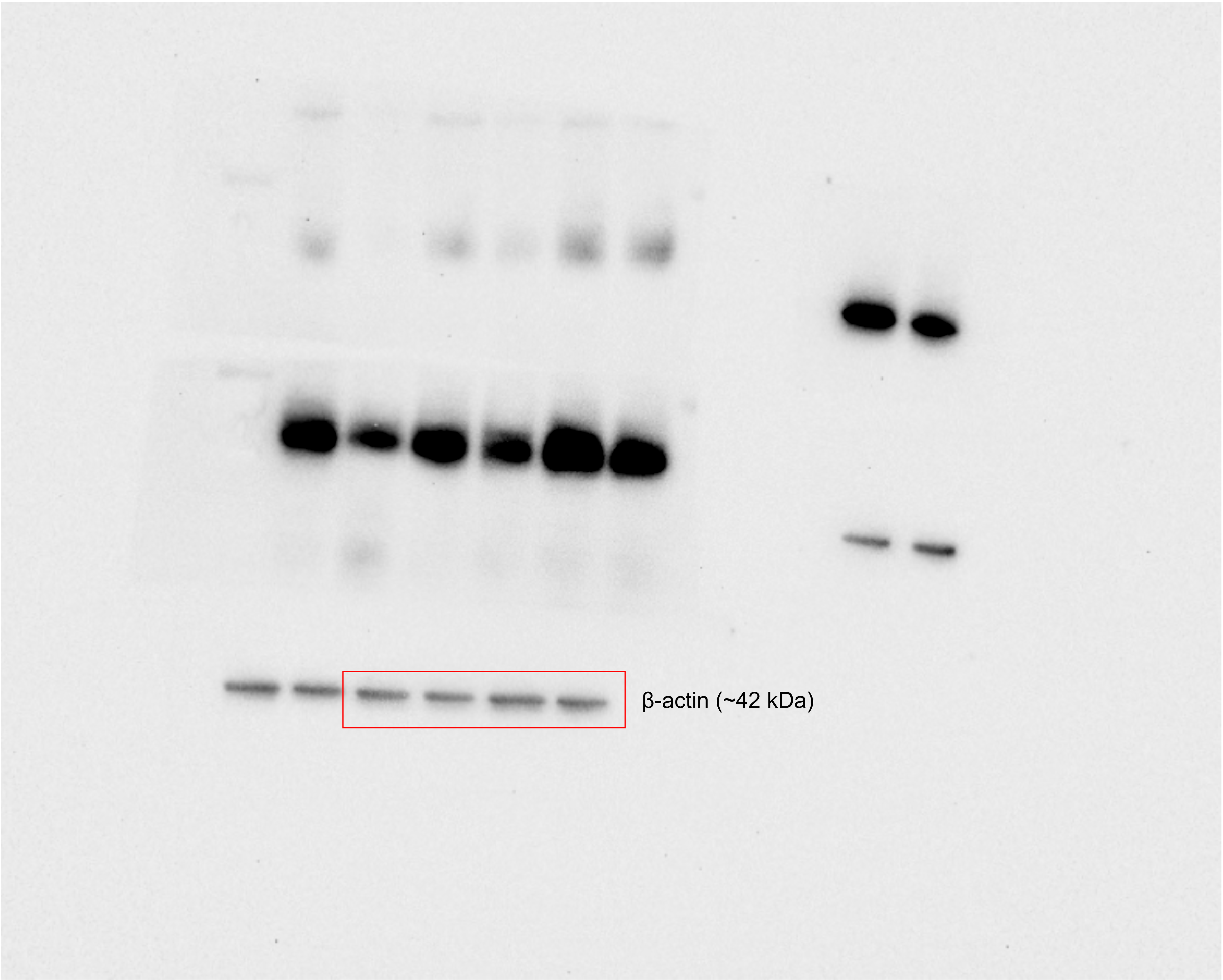

Supplement: S1 raw images — (PDF) [file pone.0352769.s005.pdf]
